# Enterocyte Proliferation and Signaling Are Constitutively Altered in Celiac Disease

**DOI:** 10.1371/journal.pone.0076006

**Published:** 2013-10-18

**Authors:** Merlin Nanayakkara, Giuliana Lania, Mariantonia Maglio, Roberta Kosova, Marco Sarno, Alessandra Gaito, Valentina Discepolo, Riccardo Troncone, Salvatore Auricchio, Renata Auricchio, Maria Vittoria Barone

**Affiliations:** 1 Department of Traslational Medicine (section of Pediatrics) and ELFID (European Laboratory for the Investigation of Food Induced Disease), University of Naples, Federico II, Naples, Italy; 2 Department of Medicine, University of Chicago, Chicago, Illinois, United States of America; Boston University Goldman School of Dental Medicine, United States of America

## Abstract

Celiac disease (CD) occurs frequently, and is caused by ingestion of prolamins from cereals in subjects with a genetic predisposition. The small intestinal damage depends on an intestinal stress/innate immune response to certain gliadin peptides (e.g., A-gliadin P31-43) in association with an adaptive immune response to other gliadin peptides (e.g., A-gliadin P57-68). Gliadin and peptide P31-43 affect epithelial growth factor receptor (EGFR) signaling and CD enterocyte proliferation. The reason why the stress/innate immune and proliferative responses to certain gliadin peptides are present in CD and not in control intestine is so far unknown. The aim of this work is to investigate if, in CD, a constitutive alteration of enterocyte proliferation and signaling exists that may represent a predisposing condition to the damaging effects of gliadin. Immunofluorescence and immunohistochemistry were used to study signaling in CD fibroblasts and intestinal biopsies. Western blot (WB) analysis, immunoprecipitation, and quantitative PCR were also used. We found in CD enterocytes enhancement of both proliferation and Epidermal Growth Factor Receptor (EGFR)/ligand system. In CD enterocytes and fibroblasts we found increase of the phosphorylated downstream signaling molecule Extracellular Signal Regulated Kinase (ERK); block of the ERK activation normalizes enterocytes proliferation in CD mucosa. In conclusion the same pathway, which gliadin and gliadin peptide P31-43 can interfere with, is constitutively altered in CD cells. This observation potentially explains the specificity of the damaging effects of certain gliadin peptides on CD intestine.

## Introduction

CD is characterized by derangement of adaptive and innate immune responses to wheat gliadins. Some gliadin peptides that are deamidated by tissue transglutaminase (e.g., A-gliadin P57-68) bind to Human Leukocyte Antigen (HLA) DQ2 and/or DQ8 molecules and induce an adaptive Th1 pro-inflammatory response [1]. Other gliadin peptides (e.g., P31-43) are able to initiate both a stress [2], [3] and an innate immune response [4], [5].

In CD, damage to the intestinal mucosa is mediated by inflammation due to both the adaptive and the innate immune responses with interleukin-15 (IL-15) as a major mediator of the innate immune response. Moreover proliferation of crypt enterocytes, causes crypts hyperplasia and mucosal remodelling, both hallmarks of CD mucosa [6], [7], [8]. In the celiac intestine, there is an inversion of the differentiation/proliferation program of the tissue. This inversion involves a reduction in the differentiated compartment that can result in complete villous atrophy and an increase in the proliferative compartment, with resultant crypt hyperplasia [9], [10].

We have previously investigated the interaction between gliadin peptides and intestinal epithelial cells in CaCo2 cells and in biopsies from CD patients. We found that P31-43 reduces the degradation of EGF Receptor (EGFR) and other Receptor Tyrosine Kinases (RTK) and prolongs their activation, which in turn results in actin modification, increased cell proliferation and other biological effects [11], [12]. Furthermore by increasing the synthesis of IL15 and the amount of the cytokine that is presented to neighbouring cells, P31-43 affects both crypts enterocyte proliferation, which is EGFR- and IL15-dependent and the activation of innate immunity [13]. The reason why the effects of these peptides are dangerous to the CD small intestinal mucosa, and not to that of controls, is not clear. Our hypothesis is that, in CD mucosa, a constitutive alteration exists, that may represent a predisposing condition to the damaging effects of gliadin. According to this hypothesis, in the present work we have attempted to determine whether constitutive alterations of signaling and proliferation occur in CD crypts enterocytes and skin-derived fibroblasts of CD patients on gluten-free diet (GFD). The alterations found are independent of the presence of gluten in the diet and of the main inflammation site.

## Materials and Methods

### Organ culture studies

For organ culture studies, biopsy fragments from duodenum were obtained from 8 CD patients with villous atrophy (mean age 5 years) 8 controls (affected by gastroesophageal reflux, (mean age 6 years) 11 CD patients on GFD (mean age 12 years) and 11 potential CD patients (mean age 7 years). GFD patients had negative serology (anti-tTg antibodies between 0 and 1,5 U/ml and EMA negative) and normal biopsy (Marsh T0-1). Potential patients had positive serology (anti-tTg antibodies between 15 and 25 U/ml and EMA positive) and normal biopsy (Marsh T0-1) [14]. Patients with villous atrophy (Marsh T3c) had positive serology (anti-tTg antibodies >50 U/ml and EMA positive). Anti-tTg antibodies were measured using Eurospital kit, EU-tTG. Informed written permission was obtained from all patients. The biopsy fragments were cultivated as reported elsewhere [11], [15]. The intestinal samples were cultured for 24 h with medium alone. The cultures were enriched with 10 µM BrdU (Sigma-Aldrich, Milan, Italy) and PD98059 [16] (Alexis Biochemicals, San Diego, USA) as required. Specimens were harvested, snap-frozen in liquid nitrogen, embedded in OCT and stored at −80°C until required.

We used double immunofluorescence to evaluate crypt proliferation in 5-µm cryostat sections from cultured biopsies [11], [15]. After a short (3 min) treatment with 1.5 N HCl, the sections were incubated with mouse monoclonal anti-BrdU (1∶150, GE Healthcare Amersham, Buckinghamshire, UK) for 1 h followed by 30 min incubation with Alexa488-conjugated anti-mouse IgG (1∶150, Invitrogen, San Giuliano Milanese, Italy) to identify BrdU-positive cells. After several washes in PBS, specimens were fixed with 3% paraformaldehyde (Sigma-Aldrich, Milan, Italy) for 5 min and incubated for 1 h with polyclonal rabbit anti-cow cytokeratin (1∶50, Dako, Milan, Italy) to stain epithelial cells. Slides were then covered for 30 min with Alexa-633-labeled goat anti-rabbit immunoglobulin (1∶200, Invitrogen, San Giuliano Milanese, Italy), contrasted with Hoechst (Sigma-Aldrich, Milan, Italy) and mounted in Mowiol 4-88. All incubations were carried out at room temperature in a dark humid chamber. The number of BrdU-positive cells divided by the total number of cytokeratin-positive cells gave the percentage of BrdU-positive cells.

### Cell culture

Fibroblasts were cultured from skin biopsies of CD patients, all of them gave informed consent to use of biopsy tissue in the study. We obtained fibroblasts from five celiac patients on gluten-free diet (age range 17–43 years) and from four HLA DQ2/8 negative healthy controls (age range 25–30 years). Patients were on gluten free diet for at least 4 years and had normal biopsy (Marsh T0), the serum levels of anti-tTg antibodies ranged between 0 and 1.6 U/ml and EMA were negative. The skin explants were immediately placed in Dulbecco's Modified Eagle's Medium (DMEM) (GIBCO, San Giuliano Milanese, Italy), 20% fetal bovine serum (FBS) (GIBCO, San Giuliano Milanese, Italy), 100 units/ml penicillin-streptomycin (GIBCO, San Giuliano Milanese, Italy), and 1 mM glutamine (GIBCO, San Giuliano Milanese, Italy) and incubated for 24 hours. Subsequently, each skin explant was divided into about 50 small fragments; these fragments were plated on Petri dishes and incubated in the presence of 95% oxygen and 5% CO2 at a temperature of 37°C to allow adhesion and subsequent release of fibroblasts. Several days later, fibroblasts began to emerge from the fragments. When fibroblasts had reached confluence, they were harvested with trypsin and frozen. In all experiments, the fibroblasts were used between the 2^nd^ and the 4^th^ passage. BrdU incorporation was performed as before [17], [18]. Briefly cells were seeded on glass coverlisps, were treated with 10 µM BrdU (Sigma-Aldrich, Milan, Italy) for 6 h then fixed for 10 min in paraformaldehyde 4% at RT, permeabilized 5 min with Triton 0.2% (Sigma-Aldrich, Milan, Italy) and treated for 3 min with 1.5 N HCl. After extensive washing the sections were incubated with mouse monoclonal anti-BrdU (1∶150, GE Healthcare Amersham, Buckinghamshire, UK) for 1 h followed by 30 min incubation with Alexa488-conjugated anti-mouse IgG (1∶150, Invitrogen, San Giuliano Milanese, Italy) to identify BrdU-positive cells. After washing the coverlips were contrasted with Hoechst (Sigma-Aldrich, Milan, Italy) and mounted in Mowiol 4-88 (Merck KGaA, Darmstadt, Germany). All incubations were carried out at room temperature in a dark humid chamber. The coverslips were observed and analyzed by a fluorescence microscope (Axiovert 2, Zeiss equipped with 40× objective, interfaced with the image analyzer software KS300). The number of BrdU-positive cells divided by the number of Hoechst positive cells gave the percentage of BrdU-positive cells.

### Phosphotyrosine staining

Fibroblasts seeded on glass coverslips were fixed with 3% paraformaldehyde (Sigma Chemical Co., Milan, Italy) for 5 min at room temperature, permeabilized with 0.2% Triton (Biorad, Milan, Italy) for 3 min at room temperature and stained for 1 h at room temperature with anti-pTyr antibody (Santa Cruz Biotechnology, CA, USA) at 1∶100. Alexa-488-conjugated secondary antibodies (Invitrogen, San Giuliano Milanese, Italy) at a dilution of 1∶100 were added to the coverslips for 1 h at room temperature in a dark chamber. Counterstaining with Phalloidin (Sigma Chemical Co., Milan Italy), was done as before [11], briefly coverlips were treated with phalloidin- Tex Red for 40 min at RT in a dark box after washing with PBS. The coverslips were then mounted on glass slides and observed by confocal microscopy (LSM 510 Zeiss). A total of 40 to 50 cells were observed in each sample, and all images were generated with the same confocal microscope. Fluorescence intensity (Fi) analysis of the samples respect to the background was carried out using AIS Zeiss software. Magnification of the micrographs is the same for all figures shown (63× objective) unless stated differently in the legends.

### Immunohistochemistry of pY-ERK in biopsies

For the immunohistochemical study, 4-µm biopsy sections were fixed in 3% paraformaldehyde (Sigma-Aldrich, Milan, Italy) for 10 min. After incubation with normal goat serum (1∶200, Dako, Copenhagen, Denmark) for 20 min, sections were covered with pY-ERK polyclonal rabbit antibodies (1∶80, Cell Signaling, Euroclone Milan, Italy) overnight. All incubations were carried out at room temperature in a humid chamber. As a negative control, some slides, were not treated with the primary antibody but with buffer solution instead. After washing with TBS (Tris- buffered solution, 0.15 M, pH 7.36, Sigma-Aldrich, Milan, Italy)+saponin (0.1%, Carlo Erba, Milan, Italy), the sections were incubated for 30 min with biotinylated goat anti-rabbit antibody (1∶300; Dako, Milan, Italy) and then with streptavidin AP (1∶400; Dako, Milan, Italy) for 30 min. New fuchsin was used as the peroxidase substrate. Finally, sections were counterstained with Mayer's hematoxylin (Sigma Diagnostic, St Louis, USA) and mounted with Aquamount (BDH, Poole, England). The preparations were analyzed using transmitted light microscopy (Nikon Eclipse 80, Nikon instruments, USA).

### Immunofluorescence for EGFR

Biopsies from CD patients with villous atrophy, GFD and controls were snap-frozen in liquid nitrogen, embedded in OCT and stored at −80°C until required. To evaluate EGFR staining 5-µm cryostat sections from biopsies were air dried and then stained with mouse anti-EGFR antibodies (F-9 sc-37707, Santa Cruz Biotechnology, CA, USA) for 1 h at room temperature, after blocking with serum bovine albumin (BSA) 3% in PBS for 30 min at room temperature (RT). Then the sections were washed (3 times, 5 min each) with PBS at RT, and secondary fluorescein conjugated anti-mouse antibody was applied for 2 h at room temperature in a dark box to avoid bleaching. After washing with PBS the slides were mounted with Mowiol and observed by fluorescent microscope (Axiovert 2, Zeiss equipped with 40× objective for interfaced with the image analyser software KS300 (Zeiss).

### EGF mRNA by laser micro dissection

The laser capture microdissection (LCM) method allows the selection of individual or clustered cells from intact tissues. Total RNA was extracted from 300 captured crypt epithelial cells from biopsies from 3 CD patients on gluten-containing diet (GCD), 3 CD patients on gluten-free diets (GFD) and 3 controls with gastroesophageal reflux. For each sample, cDNAs were transcribed using AmpliTaq Gold (Applied Biosystems, Foster City, CA). Semiquantitative PCR was carried out using oligonucleotide primers that recognize the EGF sequence: EGF, 5′-GCCAACAAACACACTGGAAA-3′ (forward) and 5′-CATGCACAAGTGTGACTGGA-3′ (reverse). The GAPDH gene was used as an example of a housekeeping gene, with the following primers: 5′-CGGAGTCAACGGATTTGGTCGTAT-3′(forward) and 5′-AGCCTTCTCCATGGTGGTGAAGAC -3′ (reverse). The PCR conditions were as follows: 1 cycle of 95°C for 10 minutes, 40 cycles of 95°C for 1 min, 60°C for 1 minute, and 72°C for 1 minute followed by 1 cycle of 72°C for 4 minutes. EGF mRNA expression has been measured selecting the number of cycles appropriated to evaluate the exponential phase of the amplification, before the reaction reaches the plateau.

### Immunoprecipitation

Cells lysates were prepared as described previously [11], [13], and protein concentration was measured using a Bio-Rad protein assay kit (Hercules, CA, USA). Equal amounts of cell lysates (2 mg protein/ml) were used for immunoprecipitation. EGFR was immunoprecipitated using anti-EGFR (Cell Signalling, Euroclone Milan, Italy). Proteins were immunoblotted with specific antibodies as described below.

### Western blotting

Briefly, fibroblast cells cultured in DMEM containing 20% FBS at 37°C were washed twice with Phosphate Buffered Saline (PBS) and resuspended in lysis buffer. Cell lysates were analyzed by SDS-PAGE and transferred to nitrocellulose membranes (Whatman Gmbh, Dassel, Germany). The membranes were blocked with 5% non-fat dry milk and probed with anti p-Tyr(P99), anti pERK, anti ERK, tubulin (Santa Cruz Biotechnology, Santa Cruz, CA, USA), and anti-EGFR (Cell Signaling EuroClone Celbio, Milan, Italy). Bands were visualized using the ECL system (GE Healthcare, Amersham, Buckinghamshire, UK). Band intensity was evaluated by integrating all the pixels of the immunostained band without the background, which was calculated as the average of the pixels surrounding the band [11], [13].

Biopsy fragments (5 mg wet weight each) from duodenum obtained from 5 CD with villous atrophy, 5 controls (affected by gastroesophageal reflux), 5 patients in remission and 5 potential CD patients were homogenized in 100 µL homogenization buffer (50 mM Tris [pH 7.4], 150 mM NaCl, 1 mM EDTA, 1 mM EGTA, 5 mM MgCl_2_, 1% TritonX100, and protease inhibitors) using a 2-mL conical Wheaton glass tube with a Teflon pestle.

### Ethical statement

The protocol of the study was approved by the Ethical Committee of the University “Federico II”, Naples, Italy (ethical approval certification C.E. n. 230/05). Informed written permission was obtained from all patients. Written informed consent was obtained from the next of kin, caretakers, or guardians on the behalf of the minors/children participants involved in our study.

## Results

### Proliferation of crypt enterocytes was increased in CD

**Figure 1:**
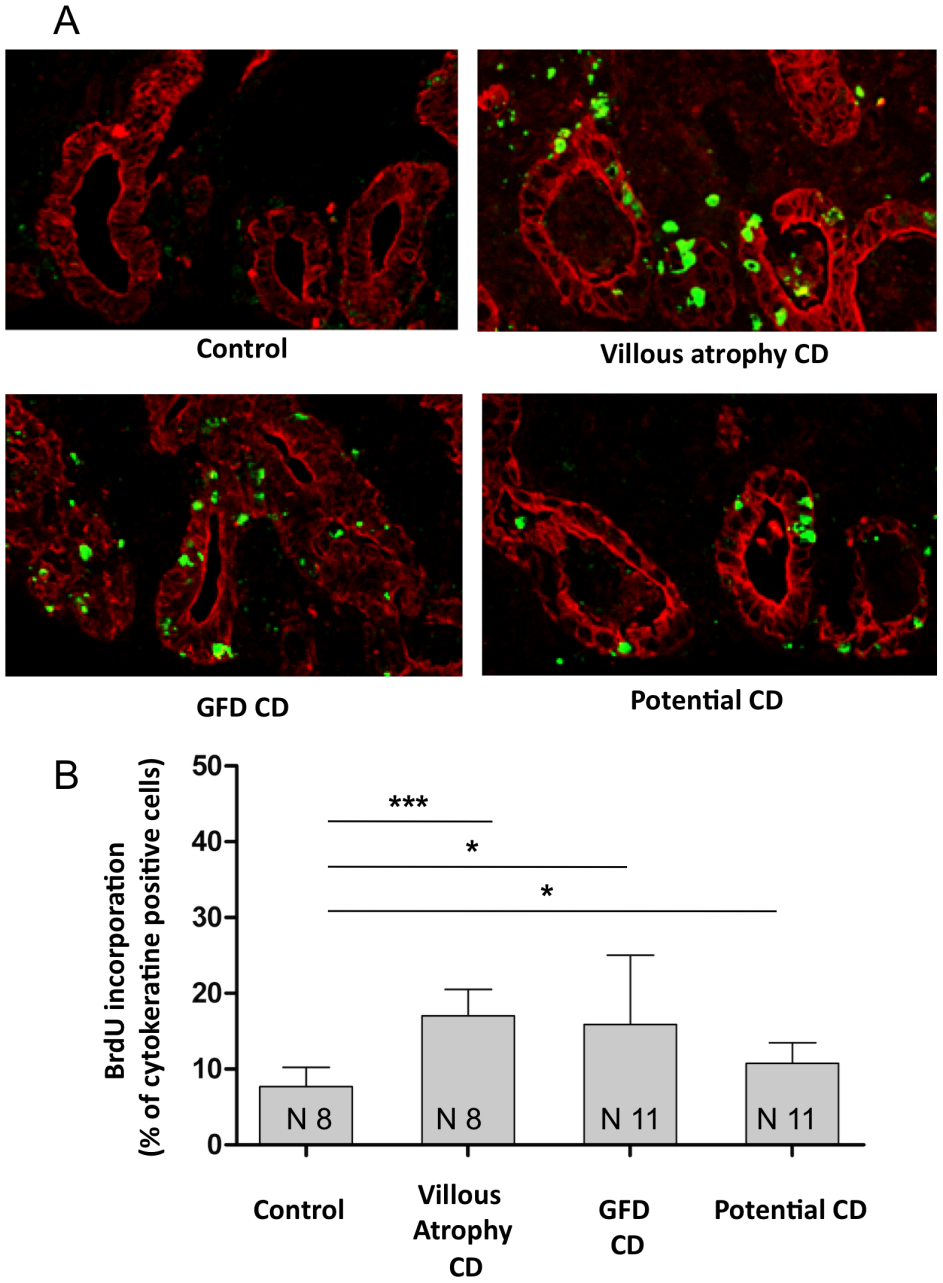
Proliferation of crypt enterocytes was increased in CD. A. Immunofluorescence images of crypts from duodenal biopsies from a control, from a CD patient with villous atrophy, from a potential CD patient who were on a gluten-containing diet and from a GFD CD patient. Biopsies were cultured for 24 h with BrdU and then stained for cytokeratin to identify epithelial cells (red) and for BrdU (green) to identify proliferating cells. One representative experiment is shown. B. Quantitation of BrdU incorporation in intestinal biopsies. More than 300 cytokeratin-positive cells were counted in several fields in each sample; the number of BrdU- positive cells was expressed as a proportion of the total cytokeratin-positive cells. Columns represent the mean, bars the standard deviation, N. is the number of biopsies tested * = P<0.05; *** = P<0.001 (Student's t-test). One-way analysis of variance (ANOVA): P value = 0.0037 (4 groups, F = 5.437, R squared = 0.3242).

We studied the proliferation of crypt enterocytes by measuring BrdU incorporation in cultured biopsies from CD patients and controls. We found that proliferation of crypts enterocytes is increased in CD patients compared to controls (figure 1 A–B). This proliferation was increased in enterocytes from patients with CD with villous atrophy (17.0%±3.5%), potential patients (10.8%±2.7%) and in CD patients in remission on a gluten-free diet (GFD) (15.9%±9.1%) with respect to controls (7.7%±2.5%). This finding indicates that the increased proliferation of crypt enterocytes seen in CD is partially independent of the crypts hyperplasia (that does not occur in potential CD) and of the presence of gluten in the diet (as it is present in patients on GFD).

### EGF mRNA and EGFR protein were increased in CD enterocytes

**Figure 2:**
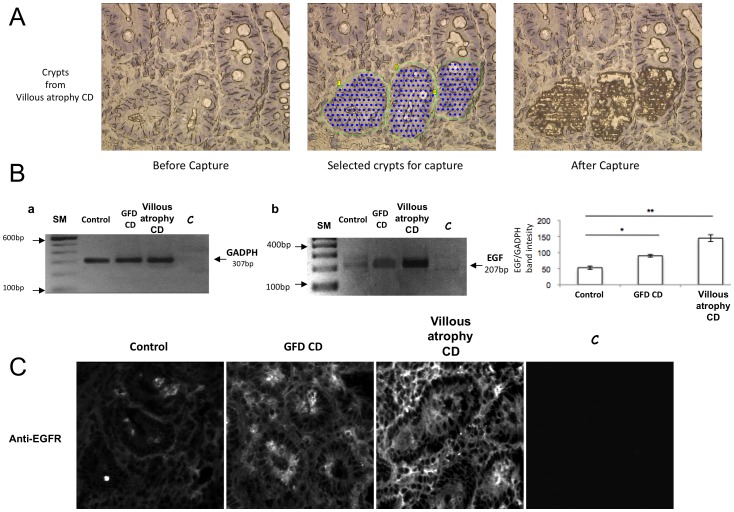
EGF mRNA and EGFR protein levels were increased in enterocytes of CD patients with villous atrophy and of GFD CD patients. Example of selected crypt enterocytes from 5-micron sections of intestinal biopsies frozen and air dried before and after capture. For each sample, 300 crypt epithelial cells were captured. B) Semiquantitative PCR analysis of a biopsy from a CD patient with villous atrophy, a CD patient on GFD and a control. (a) PCR of GADPH, used as a loading control, one representative experiment is shown, c is the control lane without mRNA. (b) PCR of EGF, one representative experiment is shown, c is the control lane without mRNA. (c) statistical analysis of data obtained from 3 CD patients with villous atrophy, 3 CD patients on gluten-free diet (GFD) and 3 controls with gastro-esophageal reflux. Columns represent the mean, bars the standard deviation, * = P<0.05; ** = P<0.01 (Student's t-test). C. Immunofluorescence of CD patients and controls' biopsies stained with anti-EGFR antibody. 40× objective. C = control without primary antibody.

We considered the possibility that in the celiac intestine proliferation of crypt enterocytes might be a possible consequence of an enhancement of the EGF/EGFR system, a potent mitogen pathway. EGF mRNA levels are generally related to the protein levels also in other systems [19]. A statistically significant increase in EGF mRNA was found, not only in enterocytes isolated by laser microdissection from biopsies of patients with CD with villous atrophy, but also in enterocytes from patients in remission on GFD (figure 2 A, B).

Moreover there was also an increase of the EGFR protein (figure 2 C) in crypts enterocytes of CD patients respect to controls. The increase was present also in GFD CD patients without crypts hyperplasia confirming that the EGF/EGFR system is enhanced in CD enterocytes independently of the gluten content of the diet and of the remodelling of the tissue.

### ERK was more phosphorylated in CD enterocytes

**Figure 3:**
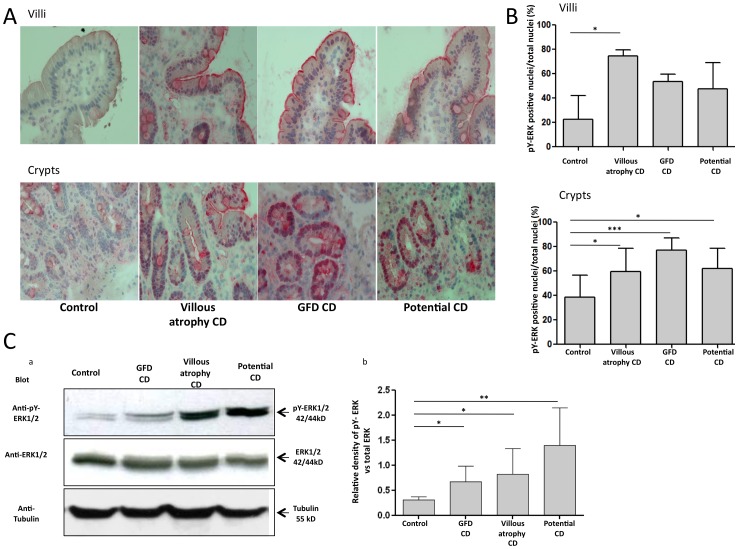
ERK was more phosphorylated in CD enterocytes. Immunohistochemical images of crypts and villi of intestinal biopsies from CD patients and controls stained with an antibody that recognizes the phosphorylated form of ERK 1/2 (pY-ERK) and with hematoxylin/eosin. One representative experiment out of 5 independent experiments is shown. B. Statistical analysis of pY-ERK positive nuclei with respect to total nuclei in the enterocytes of the crypts and villi of 5 CD patients for each group and 5 controls. More than 300 pY-ERK- positive nuclei were counted in several fields in each sample on several slides. Columns represent means and bars standard deviation. * = P<0.05; *** = P<0.001 (Student's t-test). C. (a) Western blot analysis of biopsies from CD patients and controls stained with anti-pY-ERK and anti-ERK antibodies. Similar results were obtained in 5 subjects in each group. (b) Statistical analysis of WB of biopsies from 5 subjects for each group. Columns represent the mean, bars the standard deviation of the relative intensity of pY-ERK respect to total ERK protein. * = P<0.05; ** = P<0.01 (Student's t-test).

We have tested if the downstream effector of EGFR signaling, ERK1/2 [20], [21] (figure 3 A,B,C) was activated in the celiac intestine. When ERK1/2 is activated, it migrates to the nucleus. Using an antibody against the phosphorylated form of ERK (pY-ERK), we stained biopsies from CD in the active phase of the disease (both CD with villous atrophy and potential CD) and from patients on a GFD, in the remission state of the disease. The percentage of nuclei positive for the activated form of ERK 1/2 was increased in crypt enterocytes from all CD patients (CD with villous atrophy 59.58%+/−18.86%, GFD CD 77.28%+/−9.97%, potential 69%+/−16.45%) compared to controls (38.75%+/−17.66%). In villi enterocytes, a similar trend was present but reached statistical significance only in cells derived from patients with CD with villous atrophy. Biochemical analysis of pY-ERK in biopsies from CD patients and controls confirmed the immunohystochemical analysis. As shown in figure 3C, blotting of proteins from lysates of biopsies from CD patients and controls indicated that there was a significant increase of pY-ERK not only in CD with villous atrophy and potential CD but also in CD patients on GFD. Taken together, these results indicate that ERK is activated in CD mucosa of patients whether they are on GFD or gluten containing diet (GCD).

**Figure 4:**
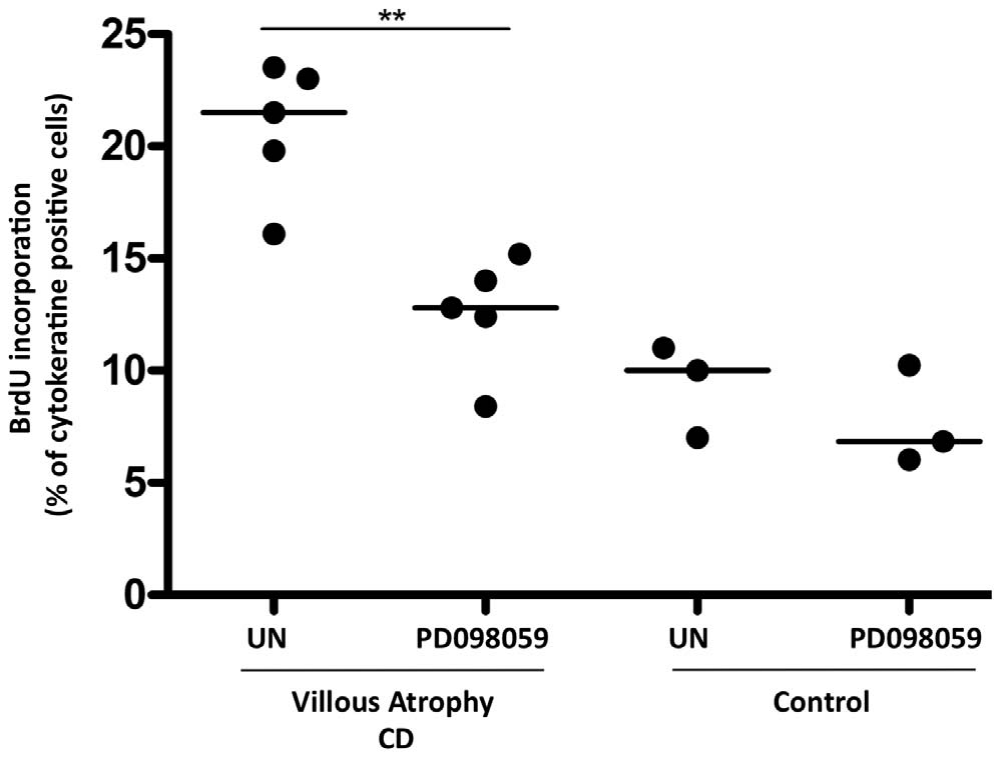
Increased proliferation of crypt enterocytes in CD depended on ERK activation. Quantitation of BrdU incorporation in intestinal biopsies. More than 300 cytokeratin-positive cells were counted in several fields in each sample; the number of BrdU- positive cells was expressed as a proportion of the total cytokeratin-positive cells. Dots represent single patients before (UN) and after ERK inhibitor PD98059, treatment. The horizontal bar is the mediane. ** = P<0.01 (Mann Whitney Test).

To investigate a link between the increased ERK phosphorylation to the increased proliferation, found in intestinal crypts epithelial cells of CD biopsies, we have blocked ERK phosphorylation by treating the intestinal biopsies in culture with a specific ERK inhibitor (PD98059) [16]. As expected, we have found that PD98059 reduced intestinal epithelial cells proliferation of the crypts enterocytes of CD mucosa with villous atrophy (% of proliferating enterocytes without PD 20.78+/−3 and with PD98059 treatment, 12.5+/−2) almost to the level found in control biopsies (% of proliferating enterocytes without PD 10+/−2) indicating that the increased proliferation of the crypts enterocytes in CD mucosa was mediated by ERK activation (figure 4). In the control biopsies in the presence of ERK inhibitor there was a tendency for a decrease of the percent of proliferating enterocytes that was not statistically significant.

### EGF mRNA, pY-ERK, pY-EGFR and total phosphorylated proteins were increased in CD fibroblasts

**Figure 5:**
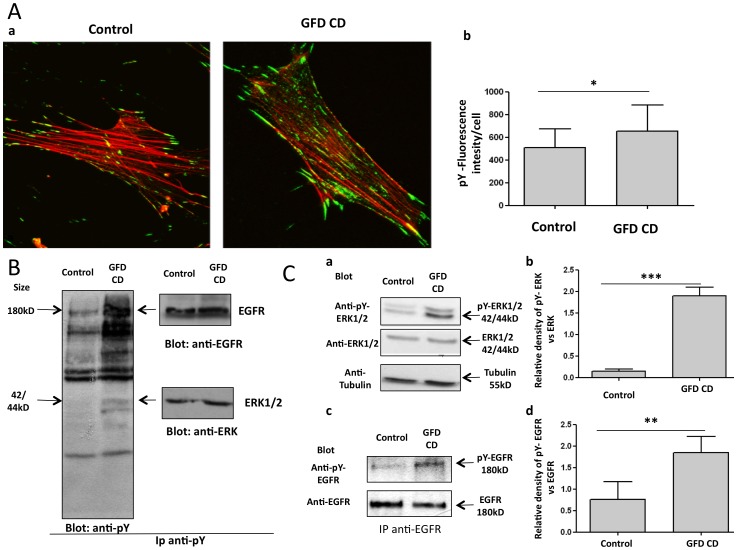
Phosphorylation of EGFR, ERK and total proteins was increased in skin fibroblasts of CD patients. Staining of total phosphorylated proteins in CD and controls fibroblasts. (a) Immunofluorescence of double staining with phalloidin (red) and anti-phosphotyrosin (green). Images obtained using a 63× objective (2× digital zoom) are shown. (b) Statistical analysis of fluorescence intensity/cell. For 5 patients and 4 controls, 3 independent experiments were done; in each experiment, the fluorescence intensity of 10 cells in random fields was measured. Columns represent means and bars standard deviation. * = P<0.05 (Student's t-test). B. Western blot analysis of total phosphorylated proteins in skin fibroblasts from CD patients on GFD and from controls. Phosphoproteins from CD patients and controls fibroblasts were lysates and immunoprecipitated (Ip), blotted and stained with anti-phosphotyrosine antibodies (blot anti-pY). The blots were stained again with anti-EGFR (blot anti-EGFR) and anti-ERK (blot anti-ERK) antibodies to identify the corresponding phosphorylated proteins. One representative experiment of 3 independent ones is shown for each subject (4 controls and 5 patients). C. Western blot analysis of phosphorylated ERK and EGFR in skin fibroblasts from CD patients on a GFD and from controls. (a) Western blot analysis of skin fibroblasts from CD patients and controls stained with anti-pY-ERK, anti-ERK and anti-tubulin antibodies. (b) Statistical analysis of WB obtained from 5 CD patients and 4 controls. Columns represent the mean, bars the standard deviation of the relative intensity of pY-ERK respect to total ERK protein. *** = P<0.001 (Student's t-test). (c) Western blot analysis of EGFR immunoprecipitated from skin fibroblasts and stained with anti-pY antibody. (d) Statistical analysis of WB obtained from 5 CD patients and 4 controls. Columns represent the mean, bars the standard deviation of the relative intensity of pY-EGFR respect to total EGFR protein** = P<0.01 (Student's t-test).

To analyze whether similar alterations were present in cells outside the intestine, we investigated the level of EGF mRNA in fibroblasts from CD patients and controls. Quantitative PCR showed that in CD fibroblasts there were significant increase of EGF mRNA levels (figure S1). Also phosphorylation of total proteins and of ERK and EGFR in skin fibroblasts from GFD CD patients and controls was studied (figure 5). Figure 5Aa shows staining for total phosphorylated proteins in fibroblasts. The fluorescence intensity/cell indicated that there were more phosphorylated proteins in fibroblasts from GFD CD patients (655.2±229.1) than in controls (510.0±164.1) (5Ab). Counter staining with phalloidin to visualize actin, identified single cells (figure 5Aa). To confirm the increment in phosphorylated proteins in CD fibroblasts, we immunoprecipitated total phosphoproteins from the cell lysate using an anti-phosphotyrosine antibody. The results, which are shown in figure 5B, demonstrated that there was an increase in the total amount of phosphorylated proteins in CD fibroblasts compared to control fibroblasts. Using specific antibodies, two of the proteins that showed increased phosphorylation were identified as ERK and EGFR.

To further confirm the increased phosphorylation of the active signaling molecules ERK and EGFR in these cells, we used western blotting to specifically analyze their phosphorylated state. As shown in figure 5Ca, pY-ERK was identified in total cell lysates of CD and control fibroblasts using specific antibodies that recognize the phosphorylated form of ERK. Densitometric analysis (figure 5Cb) showed a significant increase in the phosphorylated form of ERK in CD fibroblasts. Phosphorylated EGFR was identified by immunoprecipitating EGFR with a specific antibody and then staining the immunoprecipitated proteins with an anti-phosphotyrosine antibody (figure 5Cc). Densitometric analysis (figure 5Cd) showed an increase of almost 3-folds in the phosphorylated form of EGFR in CD fibroblasts.

Proliferation has been analyzed in fibroblasts from 4 CD patients and 5 controls. In three independent experiments for each subject we have observed increase of proliferation in CD patients fibroblasts (BrdU incorporation 31%+/−8% of the total cells) respect to controls (BrdU incorporation 25%+/−15 of the total cells) that did not reach the statistical significance.

Taken together, the data presented here imply that, in CD cells, alterations occurred in the phosphorylation of signaling proteins such as EGFR and the downstream effector ERK. These alterations were present in patients on GFD, and they were independent of the main site of inflammation.

## Discussion

In this paper, we describe constitutive alterations in enterocytes and fibroblasts of patients with celiac disease. These alterations consist of increased proliferation of crypts CD enterocytes, increased EGF mRNA and increased total phosphorylated proteins including EGFR and the downstream signaling molecule ERK. These alterations are present in patients on GFD as well as those on regular diets and, as shown by their presence in skin fibroblasts, are independent of the main inflammation site.

We measured crypts enterocytes proliferation by evaluating BrdU incorporation in organ culture experiments. As expected, crypts enterocytes proliferation was increased in active CD patients with crypts hyperplasia. Proliferation of enterocytes is a hallmark of CD during the active phase of the disease [6]–[8], [11], [13]. In potential CD patients, crypts enterocytes proliferation was also increased, although the small intestine apparently had a normal architecture in these patients. Interestingly, increased proliferation was also found in normal celiac intestine in absence of gluten from the diet in the GFD patients. Taken together, these results show that increased proliferation is an intrinsic characteristic of CD enterocytes.

Evidences from our laboratory and those of others, implicates EGFR signaling as an important pathway in celiac intestine [10], [11]. The increased proliferation of crypts enterocytes in biopsies from patients with CD with villous atrophy is dependent on EGFR and IL15 signaling. This is shown by the fact that it can be prevented by both anti-EGFR and anti-IL15 antibodies [22].

In the present work, we found that increased levels of EGF mRNA occurred not only in the enterocytes of CD patients with villous atrophy but also in patients in remission on GFD. EGF is a ligand for EGFR and the activity of the EGF receptor/ligand system depends also on the EGFR levels [19]. For this reason we have stained EGFR in CD mucosa to confirm that the EGF receptor/ligand system is activated, and we found that also the EGFR protein is increased not only in CD enterocytes with villous atrophy but also in GFD patients indicating that there is a positive autocrine loop [21] between EGFR activation and EGF mRNA production in enterocytes, that is independent of gluten intake and crypt hyperplasia.

EGFR signaling to the nuclei, to induce proliferation, is well characterized [23], [24]. Upon EGF linking to the EGFR, a signaling cascade starts, that involves sequential phosphorylation of down stream effectors such as ERK. The MAPK-ERK 1–2, like all mitogen-activated-kinases (MAPKs), is one of the essential signaling molecules that convert environmental inputs into influences on a plethora of cellular programs, including proliferation [19]. Moreover, most of the MAPK, including ERK, are stress sensors that can be activated by different inputs [20]. Only phosphorylated ERK can transduce to the nuclei, where it can start trans-activation of several genes that can induce cell proliferation and other biological effects [25], [26]. We found that more nuclei of the enterocytes from CD patients on gluten-containing diet (including patients with atrophic mucosa and potential CD patients with normal mucosa) as well as in the enterocytes of patients on GFD respect to the controls were positive for ERK, indicating that there is more ERK activity in the celiac enterocytes. To confirm the increased activation of ERK in CD mucosa we have measured the phosphorylation levels of the ERK protein and found that ERK is more phosphorylated in biopsies from CD patients with atrophic mucosa, from potential CD patients and from patients on GFD respective to the controls.

Moreover the ERK increased phosphorylation has a functional role in CD enterocytes proliferation. In fact we have found that, inhibition of ERK phosphorylation normalizes crypts enterocytes proliferation of CD atrophic mucosa indicating that the increased proliferation of celiac enterocytes is mediated by ERK activation.

ERK and EGFR activation could be an indirect effect of the residual inflammation in CD mucosa of patients at gluten free diet. For this reason we have investigated the activation of EGFR and ERK in a cellular compartment far away from the main inflammation site: the fibroblasts derived from skin of CD patients on gluten free diet. In fibroblasts from CD patients, we found an increase of phosphorylated EGFR protein confirming the activation of the EGF receptor/ligand system. Moreover we found an increase of the phosphorylated ERK in these cells, all this indicating that a signaling that involves the EGF receptor/ligand system and ERK is constitutively altered in CD cells independently from the gluten intake and in cells, such as fibroblasts, with a lower exposure to inflammation. More over we found that in fibroblasts there are other phosphorylated proteins that are increased, still to be identified.

Although CD fibroblasts have more phosphorylated proteins and increased EGF mRNA level respect to controls they show only a tendency to increase proliferation compared to control fibroblasts without reaching statistically significant differences. This apparent discrepancy could be due to several factors including the fact that primary human fibroblasts are not easily induced to proliferate, even when transformed by oncogenes they do not increase their proliferation [27]. It is also possible that in order to proliferate, together with the activation of the EGF receptor/ligand system and ERK, the CD fibroblasts need some other factor that, instead, is present in the CD intestine. Interestingly the biopsies from potential CD group have the biggest ERK activation level, but the percent of crypts epithelial cell proliferation is much less than in villous atrophy CD and GFD CD. It could be that also in this case other factors, such as cytokines, are involved in the enterocytes proliferation.

In this article we have described a CD phenotype characterized by enhancement of the EGFR/ERK pathway that could be important in the development of the disease. The EGFR/ERK pathway may have a fundamental role in the regulation of proliferation and also in the activation of the innate immunity. A role for EGF in the innate immune response has been previously described [28], [29] and previous reports suggest that airway epithelial surface signalling through EGFR is a convergent pathway resulting in innate immune responses to a variety of infectious and non-infectious noxious stimuli [30]. In CD the pro-inflammatory cytokine IL15 is a major mediator of the innate immune response [4], [5]. Interestingly EGF and IL15 cooperate to induce activation of the EGFR and IL15-R alpha pathway in CaCo2 cells, an intestinal epithelial cell line responsive to gliadin [22].

Gliadin peptides and in particular P31-43 are able to enhance proliferation of celiac enterocytes in a EGF dependent way and to delay the trafficking and degradation of EGFR at the epithelial level, suggesting a role of EGFR activation in CD, particularly in determining the crypts hyperplasia and the tissue remodelling of the CD intestine [11]. Gliadin peptides and P31-43 induced proliferation of CD crypts enterocytes (and CaCo-2 cells), is dependent not only on EGFR but also on IL15 activity [11], [13]. In particular, in CaCo-2 cells, proliferation is induced by both IL15 and EGF and is dependent by an interplay between EGFR and IL15 R-alpha: the cooperation, is mediated by a complex between the two receptors, which is activated by each ligand. P31-43, which is able to induce enterocytes proliferation, and activation of IL15 in CD, increases the complex, the activation and the downstream signalling of both receptors together with the transcripts of both ligands [22]. These data show that gliadin peptides can stimulate growth and innate immune response by using EGFR and IL15R-alpha cooperation.

In normal subjects gliadin peptide P31-43 does not induce a significant increase of proliferation in crypts enterocytes [11]–[13]. This shows that only in the celiac background gliadin is able to produce long-term damage including over-proliferation and stress/innate immune response activation.

In conclusion we looked for the presence in CD mucosa of a constitutive alteration that does not cause intestinal damage but may represent a predisposing condition to the damaging effects of gliadin. In accord with this hypothesis, we show in this paper that in celiac intestine crypts enterocytes proliferation is increased independently from the presence of gluten in the diet and that in CD cells there is an enhancement of the EGFR-ERK pathway. Thus, the same pathway that gliadin peptides can interfere with, is constitutively altered in CD cells, this potentially explaining the specificity of the damaging effects of gliadin peptides in CD.

## Supporting Information

Figure S1 **EGF mRNA is increased in Fibroblasts from CD patients respect to controls.** Quantitative PCR analysis of EGF mRNAs in fibroblasts from GFD CD patients and controls. RQ = relative quantity of mRNAs. Experiments were performed in triplicate and the data from 3 independent experiments were averaged. Columns represent the mean, and bars represent the standard deviation. * = *p*<0.05 (Student's *t*-test). cDNAs were generated from total RNA of CD GFD and controls fibroblasts, using the High Capacity cDNA Reverse Transcription Kit (Applied Biosystems, Foster City, CA, USA) [25]. The resulting cDNA samples were subjected to 10 cycles of PCR amplification followed by real-time PCR using the TaqMan® PreAmp Master Mix Kit Protocol (Applied Biosystems, PN 4366127, Foster City, CA, USA). Each TaqMan Gene Expression assay consisted of two sequence-specific PCR primers and a TaqMan assay FAM-labelled MGB probe. Eighty ng of total cDNA (as total input RNA) was used for each replicate assay, and 3 replicates were run for each sample in a 96-well plate format. Beta-2-microglobulin (B2M) was used as the endogenous control gene. Assays were run with 2× Universal PCR Master Mix without UNG (uracil-N-glycosylase) on an Applied Biosystems 7300 Real-Time PCR System using universal cycling conditions (10 min at 95°C; 15 sec at 95°C, 1 min 60°C for 40 cycles).
(DOCX)
